# The Typical tRNA Co-Expresses Multiple 5′ tRNA Halves Whose Sequences and Abundances Depend on Isodecoder and Isoacceptor and Change with Tissue Type, Cell Type, and Disease

**DOI:** 10.3390/ncrna9060069

**Published:** 2023-11-06

**Authors:** Robert Brian Akins, Kayleigh Ostberg, Tess Cherlin, Nikolas J. Tsiouplis, Phillipe Loher, Isidore Rigoutsos

**Affiliations:** Computational Medical Center, Thomas Jefferson University, Philadelphia, PA 19107, USA

**Keywords:** tRNA, tRNA halves, tRNA fragment, tRFs, short RNA, noncoding RNA, cancer, large-scale analysis, computational biology

## Abstract

Transfer RNA-derived fragments (tRFs) are noncoding RNAs that arise from either mature transfer RNAs (tRNAs) or their precursors. One important category of tRFs comprises the tRNA halves, which are generated through cleavage at the anticodon. A given tRNA typically gives rise to several co-expressed 5’-tRNA halves (5′-tRHs) that differ in the location of their 3′ ends. These 5′-tRHs, even though distinct, have traditionally been treated as indistinguishable from one another due to their near-identical sequences and lengths. We focused on co-expressed 5′-tRHs that arise from the same tRNA and systematically examined their exact sequences and abundances across 10 different human tissues. To this end, we manually curated and analyzed several hundred human RNA-seq datasets from NCBI’s Sequence Run Archive (SRA). We grouped datasets from the same tissue into their own collection and examined each group separately. We found that a given tRNA produces different groups of co-expressed 5′-tRHs in different tissues, different cell lines, and different diseases. Importantly, the co-expressed 5′-tRHs differ in their sequences, absolute abundances, and relative abundances, even among tRNAs with near-identical sequences from the same isodecoder or isoacceptor group. The findings suggest that co-expressed 5′-tRHs that are produced from the same tRNA or closely related tRNAs have distinct, context-dependent roles. Moreover, our analyses show that cell lines modeling the same tissue type and disease may not be interchangeable when it comes to experimenting with tRFs.

## 1. Introduction

Several types of short noncoding RNAs are currently known, including miRNAs and their isoforms, tRNA-derived fragments (tRFs), rRNA-derived fragments (rRFs), Piwi-interacting (piRNAs), small nucleolar RNA (snoRNAs), and Y-RNAs [1,2,3,4,5,6,7]. These molecules play important roles in cells by acting as transcription regulators, degradation mediators, and protein-binding partners [8,9,10,11]. For several of these types, their expression levels depend on “context” (e.g., tissue type, tissue state, and disease type) and “personal attributes” (e.g., sex and ancestry) [12,13,14,15,16,17]. Because of these dependencies, improving our understanding of their expression patterns and of the events in which they participate can lead to better diagnostic and therapeutic techniques [5,18,19].

**Figure 1:**
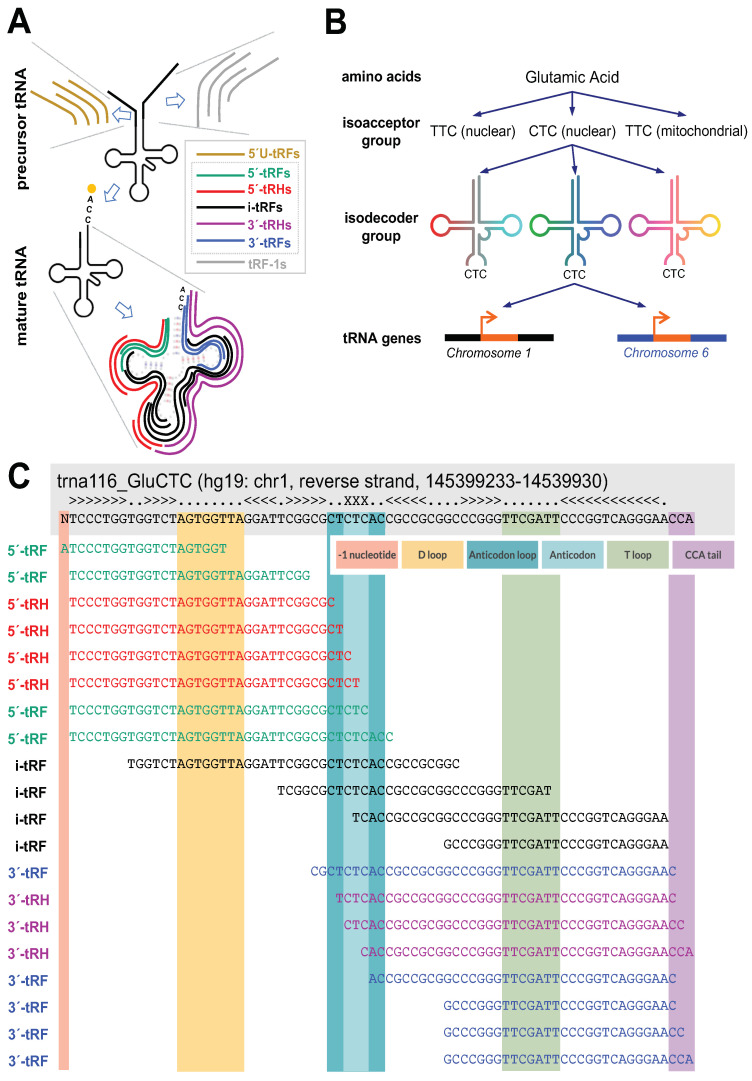
Schematic of tRF classification by length and sequence. (**A**) tRFs are separated into seven classes depending on their sequence endpoints: 5′U-tRFs, 5′-tRFs, 5′-tRHs, i-tRFs, 3′-tRHs, 3′-tRFs, and tRF-1s. 5′U-tRFs originate from tRNA precursors and begin before the tRNA coding sequence. 5′-tRFs begin at the first nt of the tRNA coding sequence or one nt before. 5′-tRHs begin at the first nt of the tRNA coding sequence or one nt before and end in one of four locations: one nt before the anticodon, at the start of the anticodon, after the first nt of the anticodon, or after the second nt of the anticodon. i-tRFs have both endpoints contained within the tRNA coding sequence excluding the terminal CCA tail. 3′-tRHs begin where 5′-tRHs end and end in any of C, CC, or CCA of the terminal CCA tail. 3′-tRFs begin anywhere in the tRNA coding sequence and end in any of C, CC, or CCA of the terminal CCA tail. tRF-1s originate from tRNA precursors and end beyond the 3′ end of the tRNA coding sequence (adapted from Figure 1 of Magee & Rigoutsos, 2020 [8], under the Creative Commons Attribution 4.0 International License). (**B**) tRFs are also classified by their tRNA source. tRNAs are grouped by the amino acids they encode. An amino acid can be encoded by multiple codons, and tRNAs whose anticodons code for the same amino acid form an isoacceptor group. A given amino acid/anticodon combination may be encoded by tRNAs found at multiple genomic loci: these tRNAs form an isodecoder group (adapted from Figure 2 of Loher et al., 2017 [36], under the Creative Commons Attribution 4.0 International License). (**C**) Examples of tRFs. tRFs are colored according to the classes to which they belong.

Concerted efforts to study tRFs began fifteen years ago with the reporting of stress-induced “5′-tRNA-halves” (5′-tRHs) and “3′-tRNA-halves” (3′-tRHs) [20,21,22]. Additional structural classes of tRFs have since been identified and described [23,24,25]. Currently, there are seven structural classes of tRFs that are distinguished by the location of their 5′ and 3′ ends within the mature tRNA or its precursor. These classes can be seen in Figure 1. The various classes of tRFs and their biogenesis, properties, and functions are reviewed elsewhere [8,25,26]. tRFs belonging to these classes can be sub-categorized further based on differences in the molecules’ terminal phosphate state compared to the typical 5′-P and 3′-OH [27].

Both the nuclear and mitochondrial genomes encode tRNAs [8,25]. tRNAs whose anticodons correspond to the same amino acid comprise an isoacceptor group. Analogously, tRNAs sharing the same anticodon belong to the isodecoder group for that anticodon. In general, for each anticodon there are multiple tRNAs encoded at different genomic locations, on the same or different chromosomes [28]. Moreover, in humans, all but one of the 22 mitochondrial tRNAs have 351 additional copies on the nuclear genomes with different degrees of sequence similarity [25].

An implicit assumption has been that tRNAs sharing the same anticodon are equivalent and, thus, interchangeable. Their multiple genomic instances have been thought to provide redundancy meant to satisfy the need for high rates of messenger RNA (mRNA) translation in response to internal and external signals. However, two lines of research suggest a more complicated situation.

First, two related studies analyzed the expression of tRNA genes from the same isodecoder in two different organisms. The first study focused on *C. elegans* and showed that eight tRNAs with identical sequences (tRNA^TrpCCA^) varied in abundance depending on tissue and time [29]. The second study focused on mice and showed that tRNAs from the same isodecoder (tRNA^PheGAA^) have distinct roles during mouse development [30]. These findings suggest that the relative abundances of tRNAs belonging to the same isodecoder, and thus the abundances of the tRFs derived from them, depend on cell state, and can have different functional roles.

Second, a study examined the relative expression of tRNAs sharing the same anticodon, and tRNAs whose anticodon corresponds to the same amino acid, in different biological samples [31]. The analyzed samples included HEK293T cells and human brain tissue. Select tRNAs from these and other cell lines and tissue samples were also examined using Northern blots. The study found that the tRNAs sharing the same anticodon exhibited tissue-dependent expression. Additionally, some of the examined samples showed differences in produced tRFs even though the corresponding tRNA levels were unchanged.

In analogy to full-length tRNAs, other studies showed differences in the levels of tRFs that are produced from different tRNAs and shorter than tRNA halves [14,23,27,32]. Larger-scale analyses also showed strong associations of tRFs shorter than halves with tissue type, disease, sex, and ancestry [15,16,25]. We are aware of only one systematic enumeration of tRNA halves [33], but it was based on datasets generated from a single cell type (lymphoblastoid cells) obtained from healthy donors to the GEUVADIS project [34]. We are not aware of an analogous effort that examined tRNA halves across different tissues and diseases. Incidentally, the datasets of The Cancer Genome Atlas (TCGA) project are not helpful in this regard because that project only reported RNAs with lengths ≤ 30 nucleotides (nts).

We set out to address this gap by investigating whether publicly available data can reveal any dependencies of tRNA halves on context. We note that we focus on public datasets that have been generated using the “standard” RNA-seq protocol and do not examine tRFs with modified ends [27,35]. Our effort builds on the work of [31] by examining the exact nucleotide sequences of 5′-tRHs that are expressed from a given tRNA, or related tRNAs (same isodecoder or isoacceptor), across tissues and diseases. To put these fragments in context, we also examined 5′-tRFs that are not “halves” but whose 3′ ends are in the vicinity of the anticodon (hereafter referred to as “long” 5′-tRFs).

## 2. Results

Using MINTmap [36], we generated the tRF profiles for 378 short RNA-seq datasets from NIH’s SRA from 10 different tissues. We analyzed each tissue separately. In these analyses, we only considered tRFs with a minimum abundance of 10 reads per million (RPM). Moreover, we sub-selected and focused only on tRFs with the following properties: their 5′ end is the same as that of the mature tRNA (or, in the case of tRNA^HisGTG^, one nt before the nominal 5′ of the mature tRNA [37,38]); and their 3′ end is in the vicinity of the anticodon, up to seven nts before or after the anticodon’s start. By definition, the sub-selected tRFs range in length between 27 and 41 nts and include 5′-tRHs. Importantly, our analyses examine only tRFs that are “visible” to the standard RNA-seq protocol, i.e., tRFs containing a 5′-P and a 3′-OH—these datasets represent the overwhelming majority of the datasets in SRA. Tables S2–S11 list the sequences and normalized abundances of all analyzed tRFs separately for each dataset.

### 2.1. The Abundances of Different-Length 5′-tRHs Depend on Context

**Figure 2:**
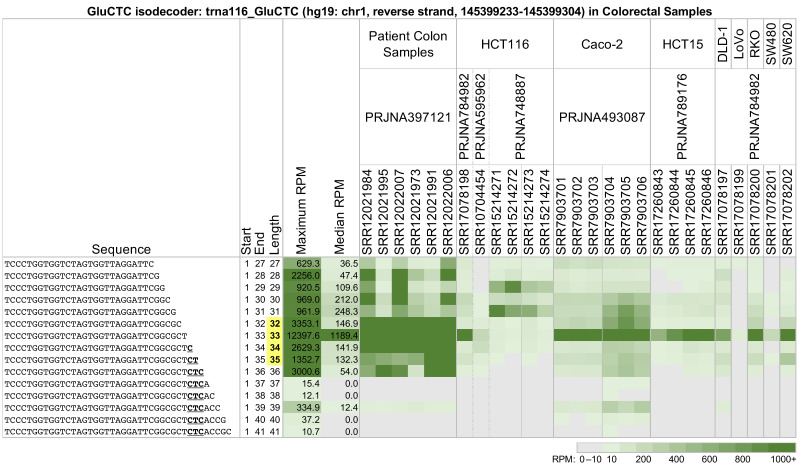
Long 5′-tRFs and 5′-tRHs from the nuclear trna116_GluCTC (hg19: chr1, reverse strand, 145399233-145399304) in colon tissues and model cell lines. In patient colon samples and colorectal cell lines, fragments vary in abundance based on their length and the nature of the biosample. Samples SRR15214273 and SRR15214274 correspond to knockdown of the RAN binding protein 1 (RANBP1). Samples SRR7903704, SRR7903705, and SRR7903706 are RIP-seq experiments using an antibody that recognizes RNAs with methylation of guanosine at position 7 (m7G). The other samples were untreated. tRFs with lengths 32–35 nts are 5′-tRHs (bold, yellow). In the sequence, the anticodon is emphasized (bold, underline). Datasets are identified by their NCBI Sequence Read Archive (SRA) bioproject (second row) and Sequence Read Run (SRR) numbers (third row). Fragments with abundance < 10 reads per million (RPM) were thresholded and are displayed as having an abundance of 0 RPM. Data coloring follows a linear scale up to 1000 RPM.

Across all analyzed datasets and tissues, we found that 5′-tRHs are not always the most abundant fragments. Figure 2 shows a heatmap of the abundance of multiple 5′-tRHs and long 5′-tRFs from trna116_GluCTC, an isodecoder of tRNA^GluCTC^ located on the reverse strand of chr1 between positions 145,399,233 and 145,399,304 (hg19 assembly coordinates). The shown profiles correspond to 27 datasets from five projects. The datasets were generated from patient colon cancer samples and the frequently used cell lines HCT116, Caco-2, HCT15, DLD-1, LoVo, RKO, SW480, and SW620.

Note how strikingly different the 5′-tRH profiles are between the colon cancer patient samples of project PRJNA397121 [39] and the colon cancer cell lines (all other projects). The richness of 5′-tRHs found in the patients is replaced by a single abundant tRNA half in the cell lines or is missing altogether; e.g., project PRJNA748887 [40].

Also notable are the changes in the abundances of tRFs as they become increasingly longer on their 3′ end. In the patient samples from project PRJNA397121 [39], the tRFs with lengths 28 or 30 nts are more abundant than those with lengths 29 or 31 nts. This pattern is reversed in the HCT116 cell line samples from project PRJNA748887 [40], with the tRFs of lengths 29 and 31 nts now being more abundant. This pattern is absent in the cell line samples from projects PRJNA784982 [41], PRJNA595962 [42], and PRJNA789176 [43].

### 2.2. tRNAs with Near-Identical Sequences from the Same Isodecoder Group Produce Different 5′-tRHs with Different Abundances

**Figure 3:**
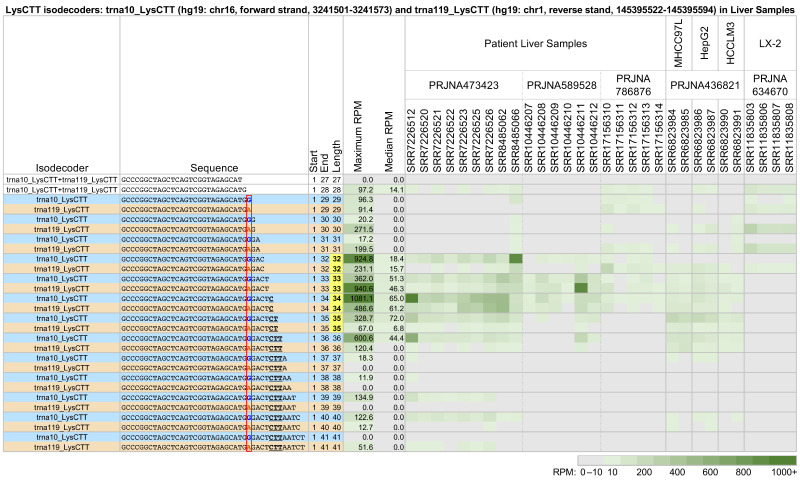
Long 5′-tRFs and 5′-tRHs from the nuclear trna10_LysCTT (hg19: chr16, forward strand, 3241501-3241573) (blue) and the nuclear trna119_LysCTT (hg19: chr1, reverse strand, 145395522-145395594) (orange) in liver tissues and model cell lines. In patient liver samples, the hepatocellular carcinoma cell lines MHCC97L, HepG2, and HCCLM3, and the hepatic stellate cell line LX-2, tRFs vary in abundance depending on their source tRNA, their length, and the nature of the biosample. Samples SRR6823984, SRR6823986, and SRR6823990 were treated with tumor-associated neutrophils (TANs). Samples SRR11835803, SRR11835804, and SRR11835805 were stimulated with TGF-ß1 for 24 h. Samples SRR11835806, SRR11835807, and SRR11835808 were stimulated with TGF-ß1 for 24 h and co-cultured with human umbilical cord mesenchymal stem cells (hUC-MSCs). The other samples were untreated. Differences in tRF sequence are bolded, colored by tRNA isodecoder, and enclosed in a red box. To enable comparisons, all other formatting, highlighting, conditional coloring, and thresholding are exactly as in Figure 2.

We also compared the tRF profiles from co-expressed tRNAs that share an anticodon and have near-identical sequences. We found that the identities and abundance of the produced fragments depend on the parental tRNA. Figure 3 shows a characteristic example of two tRNAs belonging to the LysCTT isodecoder group: trna10_LysCTT, which is located on the forward strand of chr16 between positions 3,241,501 and 3,241,573 (hg19 assembly), and trna119_LysCTT, which is located on the reverse strand of chr1 between positions 145,395,522 and 145,395,594 (hg19 assembly). These two tRNAs differ by a single nucleotide at position 29 (indicated by the red rectangle). As a result, the parental tRNA for the 5′-tRFs with lengths 27 and 28 nts is unclear. The figure shows a heatmap of all 5′-tRHs and long 5′-tRFs in 30 datasets from patient liver samples, the hepatocellular carcinoma cell lines MHCC97L, HepG2, and HCCLM3, and the hepatic stellate cell line LX-2. trna10_LysCTT has a single copy in the genome (hg19). trna119_LysCTT belongs to a group of five tRNAs with end-to-end identical sequences whose copies are spread over four chromosomes (hg19).

Each of the two tRNAs produces 5′-tRHs and long 5′-tRFs with different abundances (tRFs from each tRNA are listed on alternating lines). In the patient samples of project PRJNA473423 [44,45,46], tRNA trna10_LysCTT produces the more abundant tRFs. This is especially true for the 5′-tRHs with lengths 32–36 nts. These halves are partially absent from the patient samples of projects PRJNA589528 [47] and PRJNA786876 [48] and the cell line samples from project PRJNA436821 [49]. They are completely absent from the LX-2 cell line of project PRJNA634670 [50]. For more details, see Tables S2–S11.

### 2.3. Same-Length 5′-tRHs from tRNAs in the Same Isoacceptor Group Have Different Absolute and Relative Abundances

We also found that the absolute abundance of 5′-tRHs and long 5′-tRFs of a given length differs even for tRNAs belonging to the same isoacceptor group. Moreover, the relative abundance (that is, the ratio of abundances) of any two 5′-tRHs or long 5′-tRFs also differs in tRNAs belonging to the same isoacceptor group. Interestingly, we found that which tRNA of an isoacceptor group gives rise to the most abundant 5′-tRH or long 5′-tRF differs across datasets even when the corresponding biological samples come from the same tissue (see Tables S2–S11).

**Figure 4:**
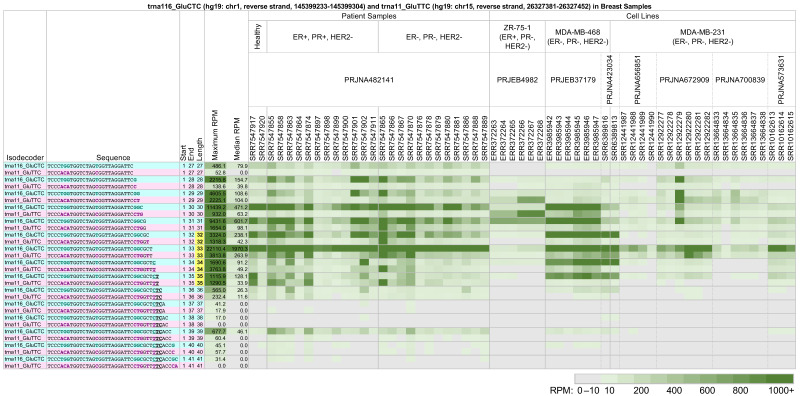
Long 5′-tRFs and 5′-tRHs from trna116_GluCTC (hg19: chr1, reverse strand, 145399233-145399304) (teal) and trna11_GluTTC (hg19: chr15, reverse strand, 26327381-26327452) (pink) in breast cancer patient samples and model cell lines. In breast cancer patient samples, the estrogen receptor-positive breast cancer cell line ZR-75-1, and the triple-negative breast cancer cell lines MDA-MB-468 and MDA-MB-231, fragments vary in abundance based on their source isoacceptor, their length, and the nature of the biosample. Samples ERR372266, ERR372267, ERR372268, ERR3985945, ERR3985946, and ERR3985947 express human estrogen receptor β (ERβ). Samples SRR12441987 and SRR12441988 were treated with 20 µM trichostatin A (TSA) for 48 h. Samples SRR12922280, SRR12922281, and SRR12922282 were transfected with the mutant retinoic acid receptor RARαS77A. Samples SRR13664833, SRR13664834, and SRR13664835 expressed control Flag vector, and samples SRR13664836, SRR13664837, andSRR13664838 expressed Flag-tagged P21-activated kinase 5 (Flag-PAK5). The other samples were untreated. Differences in tRF sequence are bolded and colored by tRNA isoacceptor. To enable comparisons, all other formatting, highlighting, conditional coloring, and thresholding are exactly as in Figure 2.

As an example, note the long 5′-tRFs and 5′-tRHs from trna116_GluCTC (see above and Figure 2) and trna11_GluTTC, located on the reverse strand of chromosome 15 between positions 26,327,381 and 26,327,452 (hg19 assembly). While these two tRNAs differ in their Glu anticodon, their nucleotide sequences are very similar. Figure 4 shows a heatmap of the 5′-tRHs and long 5′-tRFs arising from these tRNAs across 59 datasets from eight projects. The datasets correspond to biological samples generated from breast cancer patient samples, the estrogen receptor-positive breast cancer cell line ZR-75-1, and the triple-negative breast cancer cell lines MDA-MB-468 and MDA-MB-231.

In patient samples from project PRJNA482141 [51], the long 5′-tRFs and 5′-tRHs from trna116_GluCTC with lengths 30–33 nts are relatively more abundant than the corresponding fragments from trna11_GluTTC. This is true in the datasets from healthy patients and in those from patients with ER-positive, PR-positive, HER2-negative breast cancer, and triple-negative breast cancer. However, note that the dataset SRR7547864 shows similar abundances of fragments with lengths 31–33 nts from both trna116_GluCTC and trna11_GluTTC, in contrast with other datasets under the same experimental conditions in this project.

The cell line datasets examined do not show the same pattern of long 5′-tRF and 5′-tRH expression as the patient samples, or as each other. In the datasets of project PRJEB4982 [52] from the ER-positive cell line ZR-75-1, trna11_GluTTC produces more abundant long 5′-tRFs with lengths 29–30 nts than trna116_GluCTC. This is reversed in the datasets of projects PRJEB37179 [53] and PRJNA423034 [54]. In the latter two projects, trna116_GluCTC also produces more abundant 5′-tRHs than trna11_GluTTC. Also, in the datasets of project PRJNA573631 [55,56], only the 5′-tRH with length 33 nts from trna116_GluCTC shows higher abundance than its counterpart trna11_GluTTC. Lastly, note the similarities and differences in the 5′-tRHs and long 5′-tRFs that are produced from trna116_GluCTC and trna11_GluTTC in the MDA-MB-231 datasets of projects PRJNA656851 [57], PRJNA672909 [58], and PRJNA700839 [59].

### 2.4. The Same tRNA Can Produce Different 5′-tRHs with Different Relative Abundances in Tissues and Cell Lines from the Same Tissue

**Figure 5:**
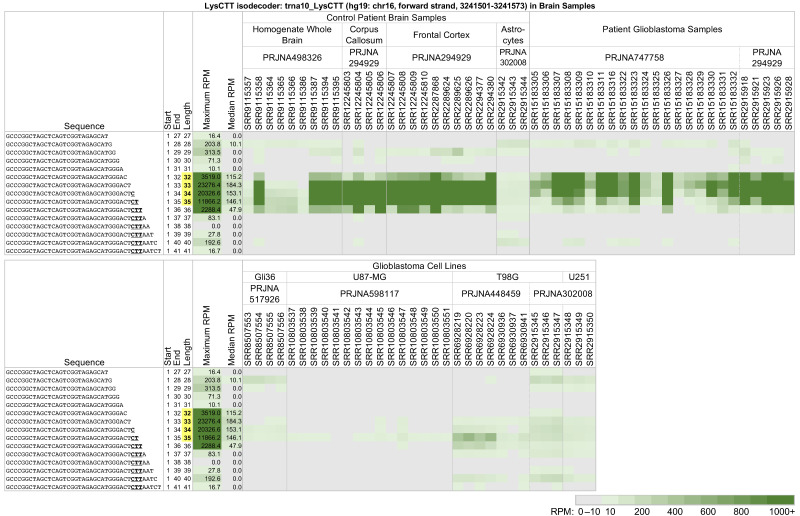
Long 5′-tRFs and 5′-tRHs from the nuclear trna10_LysCTT (hg19: chr16, forward strand, 3241501-3241573) in brain samples and glioblastoma model cell lines. In control patient brain samples, patient glioblastoma samples, and the glioblastoma cell lines Gli36, U87-MG, T98G, and U251, tRFs SRR10803543, SRR10803544, and SRR10803545 were subjected to hypoxia and Hif1a knock-out. Samples SRR10803546, SRR10803547, and SRR10803548 were subjected to hypoxia and Hif2a knock-out. Samples SRR10803549, SRR10803550, and SRR10803551 were subjected to hypoxia, Hif1a knock-out, and Hif2a knock-out. Samples SRR6928219 and SRR6928223 were treated with EZH2 knock-out and VP55 overexpression. Samples SRR6928220 and SRR6928224 were treated with VP55 overexpression. Samples SRR6928236 and SRR6928237 were treated with EZH2 knock-out. The other samples were untreated. To enable comparisons, formatting, highlighting, conditional coloring, and thresholding are exactly as in Figure 2.

Figure 2 and Figure 4 showed cases of tRFs where the relative and absolute tRF abundance varied between tissue samples and cell lines from the same tissue. We investigate the matter further by analyzing 5′-tRHs and long 5′-tRFs from a single tRNA across three different sources: control brain samples, glioblastoma samples, and the glioblastoma cell lines Gli36, U87-MG, T98G, and U251 (Figure 5). We found that trna10_LysCTT (see above) produces a very different collection of 5′-tRHs and long 5′-tRFs in the patient samples compared to the glioblastoma cell lines.

Among the brain samples, the 5′-tRHs of trna10_LysCTT are most abundant in the control samples—projects PRJNA498326 [60] and PRJNA294929 [61]—and the glioblastoma patient samples—projects PRJNA747758 [62] and PRJNA294929. In contrast, the glioblastoma cell line samples from projects PRJNA517926 [63], PRJNA598117 [64,65], PRJNA448459 [66], and PRJNA302008 [67] produce only some of these fragments, if at all, and at a much lower abundance. It is worth noting that in liver patient samples and cell lines, this tRNA produces a more diverse collection of considerably less abundant fragments (see Figure 3).

### 2.5. The Identities and Abundances of 5′-tRHs from a Given tRNA Differ Significantly among Cell Lines from the Same Tissue

We also examined how the profiles of fragments with lengths between 27 and 41 nts change across cell lines of the same tissue. As seen in Figure 2, trna116_GluCTC produces primarily a 34 nt long 5′-tRH in the cell lines Caco-2, HCT15, DLD-1, RKO, and SW620. In the dataset SRR17078198 from the cell line HCT116, trna116_GluCTC produces primarily the same 34 nt long fragment. However, in the HCT116-derived datasets SRR15214271, SRR15214272, SRR15214273, and SRR15214274, the same tRNA produces two long 5′-tRFs (29 nts and 31 nts). Finally, in the HCT116-derived SRR10704454, the LoVo-derived SRR17078199, and the SW480-derived SRR17078201, the production of 5′-tRHs and long 5′-tRFs from trna116_GluCTC is comparatively subdued.

We can see a similar pattern in the liver samples of Figure 3 as well. Both trna119_LysCTT and trna10_LysCTT produce fragments with lengths 33–36 nts in the liver cancer cell lines MHCC97L, HepG2, and HCCLM3. In contrast, in the hepatic stellate cell line LX-2, neither trna119_LysCTT nor trna10_LysCTT produce any fragments longer than 32 nts.

Pronounced differences in the expression of 5′-tRHs and long 5′-tRFs can be seen even when the cell lines model the same disease subtype. For example, in Figure 4, we presented data from the MDA-MB-468 and MDA-MB-231 cell lines, which model triple-negative breast cancer. As can be seen, trna116_GluCTC’s production of 5′-tRHs and long 5′-tRFs differs markedly between the cell lines. In MDA-MB-468, there is a clear enrichment of fragments with lengths 30–35 nts. These fragments are missing from MDA-MB-231, except for the 33 nt long 5′-tRH in samples SRR6389813 and SRR12922279.

Lastly, we note the dataset SRR6389813 from PRJNA423034 [54]. While it is derived from MDA-MB-231, its tRF profile closely resembles that of SRR6389816 from the same project, the latter derived from MDA-MB-468.

## 3. Discussion

We analyzed hundreds of public datasets derived from biological samples from 10 different tissues. To ensure the biological origin of each dataset, we manually examined the information that accompanies each dataset. Our analyses focused on 5′-tRHs and “long” 5′ tRFs whose 3′ endpoints are in the vicinity of the anticodon. While there are many large studies of tRFs, they have focused on fragments shorter than the ones we discussed here. We are not aware of any systematic analyses of deep sequencing datasets that investigated 5′-tRHs or long 5′ tRFs and their possible dependence on tRNA with respect to origin and context.

Our analyses led to several observations, including:

- The exact endpoints and absolute and relative abundances of 5′-tRHs and long 5′-tRFs depend on the identity of the parental tRNA.
- The exact endpoints and absolute and relative abundances of 5′-tRHs and long 5′-tRFs also depend on cell/tissue type and disease.
- tRNAs with highly similar sequences that belong to the same isodecoder will, in general, produce different groups of 5′-tRHs and long 5′-tRFs in the same tissue.
- tRNAs with highly similar sequences that belong to the same isoacceptor will, in general, produce different groups of 5′-tRHs and long 5′-tRFs in the same tissue.
- A given tRNA could produce no 5′-tRHs, no long 5′-tRFs, or neither 5′-tRHs nor long 5′-tRFs.
- As a group, long 5′-tRFs have different and generally lower abundances than 5′ tRHs.

Next, we discuss these and other findings along with their implications. Before proceeding, we stress that our analyses and the resulting observations refer only to tRFs with 5′-P and 3′-OH termini, a combination that is assumed by the standard RNA sequencing protocols. Such datasets represent the overwhelming majority of datasets in NIH’s SRA repository. Indeed, profiling molecules with terminal states other than 5′-P/3′-OH requires modified sequencing protocols; as a result, few such datasets currently exist in NIH’s SRA repository.

It is important to note that if standard deep sequencing does not show any halves being produced by a tRNA, this does not automatically mean that the tRNA produces halves with modified termini. Indeed, one of the earliest studies in the field used Northern blots, which capture tRFs with both modified and unmodified termini, and showed that the same abundant tRNA (tRNA^GlyGCC^) produced halves with widely varying abundances in different cell lines [22]. The same study showed for another tRNA (tRNA^ArgACG^) that the amount of tRNA halves it produced depended on the length of time cells were subjected to starvation. Yet another tRNA (tRNA^TyrGTA^) never produced halves, regardless of the length of starvation time.

Our study placed emphasis on examining 5′-tRHs. For completeness, we also considered long 5′-tRFs with 3′ ends up to seven nts before or after the start of the anticodon. Because of their common 5′ end and near-similar sequences and lengths, these fragments have traditionally been treated together without any systematic attempt to determine whether they are distinguishable from one another.

Compared to shorter fragments that have a rich diversity of 5′ and 3′ endpoints, tRNA halves are limited: they are produced through cleavage of the mature tRNA in the vicinity of the anticodon. Initially, it was believed that the ribonuclease Angiogenin (ANG) produced all tRNA halves [22,68]. However, recent work showed that ANG is responsible for producing tRNA halves from only seven isoacceptors [69]. Members of the RNase A superfamily can also produce tRNA halves in the absence of ANG [70], whereas RNase L produces tRNA halves from specific tRNAs in response to viral infection [71]. Additionally, RNase Z/ELAC2 and Dicer have also been implicated in the production of tRNA fragments. The RNase Z/ELAC2 has been shown to produce tRFs from the 3′ trailers of tRNAs in human cell lines [23,72]. The situation with Dicer is more complicated in that tRF production has been shown to both depend [73,74,75] and not depend [32,76] on it. Lastly, Schlafen family member 11 (SLFN11) has also been shown to cleave tRNAs with a long variable loop in response to DNA damage [77].

The complexity in the identities and abundances of the produced 5′-tRHs and long 5′-tRFs that our analyses unraveled likely reflects the aggregate effects of different combinations of ribonucleases as well as contributions from additional factors. One factor that is frequently suggested relates to base modifications and their potential impact on the observed tRF endpoints. However, our large-scale analysis of numerous samples from 32 cancer types [15] found no correlation between the location of base modifications and the endpoints of the produced tRFs. A second, and perhaps more likely, contributing factor relates to the personal attributes of the patients. These include a person’s sex and ancestry, both of which have been linked to differences in tRF expression [13,15,16,25]. Age likely represents an additional dependency, but the publicly available datasets do not currently allow this determination. Lastly, it remains formally possible that differences in sample preparation and library preparation kit choices may influence the sequence range and abundance of the tRFs we observe when comparing datasets generated from different groups.

Notwithstanding the factors responsible for the observed complexities, there is clear consistency in the profiles we observe among similar samples, even when those are generated by different groups. Thus, it is reasonable to posit that the findings suggest distinct functions for each of the tRNA halves and long 5′-tRFs, even though they may differ by one or two nucleotides from one another. Indeed, several additional findings support this possibility. It has been shown that 5′-tRFs and 3′-tRFs are loaded on Argonaute and act like miRNAs [32]. This suggests that 3′-tRFs with different 5′ ends have different “seeds” and, thus, may have different targets [7]. It is worth noting here that miRNAs are known to exhibit variability in their length, typically spanning 18–22 nts. More recently, it became apparent that 3′ non-templated isomiRs also exist, are abundant, and their length generally exceeds that of the typical isomiR [78]. This 3′ richness is consequential based on previous reports that the 3′ ends of miRNAs are target-determining [79,80]. Considering how 5′-tRFs also show extensive 3′ variations, these miRNA/isomiR findings suggest that 5′-tRFs with different 3′ ends may have different targets as well. We also note that tRFs have been shown to have other functions unrelated to their entering the RNA interference pathway. These are reviewed in [8] and include direct interactions with other RNA-binding proteins like YBX1, nucleolin, and reverse transcriptase; mediation of intergenerational small RNA inheritance; direct RNA cleavage through RNAse Z(L) interactions; stabilization of mRNAs; and inhibition of protein synthesis.

One of the notable observations resulting from our analyses is that the abundance of long 5′-tRFs and 5′-tRHs depends on length: tRFs that differ by a single nt in their 3′ ends can have very different abundances. Figure 2 highlights these differences for several tRFs from trna116_GluCTC: in some of the samples, it is the 5′-tRFs with lengths 28 and 30 nts that are most abundant, whereas in other samples, it is the 5′-tRFs with lengths 29 and 31 nts. These differences extend to other tRNAs (see Tables S2–S11). We note that the differences in the abundance of 5′-tRFs which differ by a single nt in their 3′ ends are not unique to long 5′-tRFs and 5′-tRHs: they were previously reported for shorter (18–24 nts) tRFs from the nuclear-encoded tRNA^HisGTG^. Unlike the cases we discussed here, and in addition to the absolute abundance of these shorter 5′-tRFs changing with the length of the tRFs, their abundance ratios were exceptionally well-conserved across multiple tissues, in health and in disease [8,15].

Since the tRNAs mentioned above are co-expressed, the observed differences do not result from differential DNA accessibility at the corresponding loci. By also considering the observed variations in abundance ratios of different tRF pairs from the same parental RNA, we conclude that the uncovered differences likely result from differential processing of the full length tRNA. The rules that govern this processing are currently unknown and differ between patient samples and model cell lines. And as we have already shown, the rules also differ across tissues [15] and depend on personal attributes [15,16,25].

Another notable result from our analysis is the demonstration that tRNAs belonging to the same isodecoders or the same isoacceptors can generate very different tRF profiles, even when their respective sequences are highly similar. Figure 3 shows one such example with the help of two tRNAs from the tRNA^LysCTT^ isodecoder, trna10_LysCTT and trna119_LysCTT, and liver samples. The two tRNAs, which differ by a single nt at position 29, produce tRFs in very different combinations and at different levels of abundance. Figure 4 shows another example using two tRNAs belonging to the same isoacceptor (Glu): trna116_GluCTC and trna11_GluTTC, and breast cancer samples. While the two isoacceptors share very similar sequences, the profile of the tRFs they produce differs extensively in both the patient samples and the cell lines.

Looking at Figure 3 and Figure 4, we see that the abundances of the produced fragments in each case are comparable. This would suggest comparable levels of expression for the respective parental tRNAs. However, in each case, we see very clear differences in the actual fragments that are produced. The case of Figure 3 is particularly notable because the two parental tRNAs belong to the same isodecoder and differ by a single nt.

Related to this, it was recently shown that tRNAs from the same isodecoder (tRNA^PheGAA^) have functionally distinct roles in mouse development [30]. In that study, mice lacking specific tRNA loci belonging to the tRNA^PheGAA^ isodecoder showed reduced viability due to amino acid misincorporation. Along with protein translation, the control of tRF production that is suggested by our analyses provides another mechanism for functional consequences associated with tRFs from tRNAs belonging to the same isodecoder.

These observations add to the presumed complexity of the biogenesis rules and can also pose a problem for experimental procedures that rely on hybridization, such as Northern blots. Depending on the sequence specifics of the tRNA of interest, a probe sequence aimed at validating the presence of a tRF from a given tRNA may hybridize to more than just the intended target. For example, a probe aimed at position 20 through 39 of trna10_LysCTT will not be able to distinguish between fragments that arise from trna10_LysCTT or trna119_LysCTT and will hybridize to both. The situation becomes more complicated when the same anticodon is shared by many tRNAs with different pair-wise similarities, as is the case with tRNA^AspGTC^ [36].

Generally, we find that there are more, and more abundant, long 5′-tRFs whose 3′ ends are before the anticodon than after. However, we also encountered notable exceptions, like the fragments of trna10_LysCTT and trna119_LysCTT in patient liver samples from PRJNA473423, where fragments that extend past the anticodon are more abundant (Figure 3).

The abundance profiles of 5′-tRFs that result from cleavage after the anticodon are not homogeneous. Long 5′-tRFs whose last nucleotide is the last position of the anticodon exhibit abundance patterns that are more similar to those of 5′-tRHs than to other long 5′-tRFs that extend past the anticodon. This can be seen in patient samples from projects PRJNA397121 (Figure 2), PRJNA473423 (Figure 3), and PRJNA498326 and PRJNA294929 (Figure 5). 5′-tRFs whose 3′ ends are beyond that position exhibit more diverse profiles and are less abundant (Figure 2, Figure 3 and Figure 4).

Typically, 5′-tRHs are more abundant than the long 5′-tRFs that surround them. The abundance ratios of different 5′-tRHs are not constant but a function of tissue (cf., Figure 2, Figure 3 and Figure 4). It also changes across patient samples from the same tissue/disease combination. If fact, in some instances, the differences in abundance ratios of tRF pairs across patients can be very pronounced. For example, in some glioblastoma patient samples from project PRJNA747758, isodecoder trna10_LysCTT produces 5′-tRHs that have a more than ten times greater normalized abundance than in the rest of the samples (Figure 5).

Our analysis also identified pronounced differences in the expression of 5′-tRHs and long 5′-tRFs between patient samples and cell lines modeling the respective disease. Figure 4 and Figure 5 offer two characteristic examples. In Figure 4, we juxtaposed tRF expression patterns of long 5′-tRFs and 5′-tRHs from the tRNAs trna116_GluCTC and trna11_GluTTC (Glu isoacceptor) in breast cancer patients and several model cell lines. The diverse fragmentation pattern found in the triple-negative breast cancer patient samples is not recapitulated by any of the MDA-MB-231 samples. The fragmentation pattern and abundance ratios observed in the MDA-MB-468 model cell lines are closer to those of the patients. Still, this model cell line also exhibits notable differences from the patient samples. Note that ten samples from these cell lines (SRR12441987, SRR12441988, SRR12441989, SRR12441990, SRR12922280, SRR12922281, SRR12922282, SRR13664836, SRR13664837, and SRR13664838) were treated with experimental conditions, but even the control runs do not share the patient profile. In Figure 5, we juxtaposed expression patterns from the tRNA trna10_LysCTT in glioblastoma patients and the glioblastoma model cell lines Gli36, U87-MG, T98G, and U251. Again, in all four cell lines, the 5′-tRHs and long 5′-tRFs from this tRNA have much lower abundance than the glioblastoma patient samples. In fact, the expression profiles across all four cell lines are a better match for the control astrocyte samples from project PRJNA302008. Note that 15 of these glioblastoma cell line samples (SRR10803543 to SRR10803551, SRR6928219, SRR6928220, SRR6928223, SRR6928224, SRR6928236, and SRR6928237) are under experimental conditions and that the control samples still do not share the patient profile.

The differences we observed between patient samples and model cell lines, in glioblastoma and triple-negative breast cancer, were based on fragments from only a handful of tRNA sequences. Moreover, the comparisons pitted bulk tissue on one hand with a cell line on the other. Even so, based on the diversity of datasets that we analyzed, the data strongly suggest that the differences extend to many more settings than those we analyzed. This is supported by the very extensive differences between MDA-MB-231 and MDA-MB-468, two popular cell lines that model triple-negative breast cancer (Figure 4).

Taken together, our findings lead to several conclusions. First, despite their seeming redundancy, tRNAs belonging to the same isodecoder group or the same isoacceptor group have different roles, and these differences extend to the tRFs that the tRNAs produce. Second, it appears that even single nt differences among 5′-tRHs and long 5′-tRFs from the same tRNAs, tRNAs sharing an anticodon, or tRNAs whose anticodons encode the same amino acid are meaningful: while the levels and relative abundances of the corresponding fragments mirror themselves in like samples, they can differ radically in different tissues and across diseases. Third, our analyses show that cell lines modeling the same tissue type and disease may not be interchangeable when it comes to experimenting with tRFs. Fourth, analyses of bulk tissue may identify potentially causal tRFs whose functions may be difficult to study because they are not recapitulated in commercially available model cell lines. Future research into the roles of tRFs in model cell lines requires careful analysis using the data collected here or from other sources to ensure that the tRFs are accurately represented. We conjecture that these conclusions extend to the experimental study of isomiRs [16,81] and rRFs [2,17], given the previously documented variations in the abundances of these RNA fragments as well.

## 4. Materials and Methods

### 4.1. Data Selection

We identified and analyzed 378 short RNA-seq datasets from NIH’s Sequence Read Archive (SRA). Table S1 lists the Sequence Read Run (SRR) identifiers for these datasets. The datasets represent ten tissues: brain, breast, colorectal, fibroblast, kidney, liver, lung, ovary, prostate, and skin. Each dataset contains reads with lengths ≥ 50 nts. We manually screened the online information for each dataset to ensure the provenance of the biological sample (source tissue/cell type and disease state, as applicable).

### 4.2. Data Acquisition and Processing

We downloaded the fastq files from NIH’s SRA repository. The relevant accession numbers are listed in the respective subsections. We quality-trimmed reads and removed adapters using *cutadapt* [82] with default settings. We processed the cleaned-up reads with MINTmap [36] to generate the corresponding tRF profiles. MINTmap is an ideal choice for this task because it is deterministic and guarantees the exhaustive identification of tRFs in the processed FASTQ files. Moreover, MINTmap identifies and reports only tRFs that match the known tRNAs exactly (i.e., no replacements or indels). For tRFs that may be present in multiple tRNAs [36], MINTmap unambiguously calculates and reports their (raw and normalized) abundances while avoiding double-counting. Lastly, and in addition to being more sensitive and specific than other tools [36], MINTmap identifies and “flags” as candidate false positives those tRFs that exist in parts of the genome that are unrelated to tRNAs.

## Data Availability

All data accession information can be found in Table S1.

## Supplementary Materials

The following supporting information can be downloaded at https://www.mdpi.com/article/10.3390/ncrna9060069/s1. Table S1, Analyzed datasets sorted alphabetically by tissue label, bioproject number, and Sequence Read Run (SRR) number; Table S2, Long 5′-tRFs and 5′-tRHs from analyzed brain tissue data; Table S3, Long 5′-tRFs and 5′-tRHs from analyzed breast tissue data; Table S4, Long 5′-tRFs and 5′-tRHs from analyzed colorectal tissue data; Table S5, Long 5′-tRFs and 5′-tRHs from analyzed fibroblast tissue data; Table S6, Long 5′-tRFs and 5′-tRHs from analyzed kidney tissue data; Table S7, Long 5′-tRFs and 5′-tRHs from analyzed liver tissue data; Table S8, Long 5′-tRFs and 5′-tRHs from analyzed lung tissue data; Table S9, Long 5′-tRFs and 5′-tRHs from analyzed ovary tissue data; Table S10, Long 5′-tRFs and 5′-tRHs from analyzed prostate tissue data; Table S11, Long 5′-tRFs and 5′-tRHs from analyzed skin tissue data.
